# Gibberellin Induced Transcriptome Profiles Reveal Gene Regulation of Loquat Flowering

**DOI:** 10.3389/fgene.2021.703688

**Published:** 2021-09-10

**Authors:** Yuanyuan Jiang, Yicun Liu, Yongshun Gao, Jiangrong Peng, Wenbing Su, Yuan Yuan, Xianghui Yang, Chongbin Zhao, Man Wang, Shunquan Lin, Ze Peng, Fangfang Xie

**Affiliations:** ^1^Henry Fok College of Biology and Agriculture, Shaoguan University, Shaoguan, China; ^2^State Key Laboratory for Conservation and Utilization of Subtropical Agro-Bioresources, College of Horticulture, South China Agricultural University, Guangzhou, China; ^3^College of Agriculture and Landscape Architecture, Zhongkai University of Agriculture and Engineering, Guangzhou, China; ^4^Beijing Academy of Forestry and Pomology Sciences, Beijing, China; ^5^Beijing Engineering Research Center for Strawberry, Beijing, China; ^6^Fruit Research Institute, Fujian Academy of Agricultural Science, Fuzhou, China

**Keywords:** GA_3_, flowering, RNA-seq, co-expression, loquat

## Abstract

Flowering is an integral part of the life cycle of flowering plants, which is essential for plant survival and crop production. Most woody fruit trees such as apples and pears bloom in spring, but loquat blooms in autumn and winter. Gibberellin (GA) plays a key role in the regulation of plant flower formation. In this study, we sprayed loquat plants with exogenous GA_3_, which resulted in vigorous vegetative growth rather than floral bud formation. We then performed a comprehensive RNA-seq analysis on GA_3_-treated and control-treated leaves and buds over three time periods to observe the effects of exogenous GA_3_ application on floral initiation and development. The results showed that 111 differentially expressed genes (DEGs) and 563 DEGs were down-regulated, and 151 DEGs and 506 DEGs were up-regulated in buds and leaves, respectively, upon treatment with GA_3_. Among those that are homologs of the DELLA-mediated GA signal pathway genes, some may be involved in the positive regulation of flower development, including *EjWRKY75, EjFT, EjSOC1, EjAGL24, EjSPL, EjLFY, EjFUL*, and *EjAP1*; while some may be involved in the negative regulation of flower development, including *EjDELLA, EjMYC3, EjWRKY12*, and *EjWRKY13*. Finally, by analyzing the co-expression of DEGs and key floral genes *EjSOC1s, EjLFYs, EjFULs, EjAP1s*, 330 candidate genes that may be involved in the regulation of loquat flowering were screened. These genes belong to 74 gene families, including Cyclin_C, Histone, Kinesin, Lipase_GDSL, MYB, P450, Pkinase, Tubulin, and ZF-HD_dimer gene families. These findings provide new insights into the regulation mechanism of loquat flowering.

## Introduction

The floral transformation of plants is affected by various endogenous and exogenous factors, forming a very sophisticated and complex regulatory network. It can accurately respond to internal and external signals and integrate them together to ensure that plants bloom at a favorable time and reproduce successfully. Plants can accurately sense photoperiod changes and adjust flowering time (Shim et al., 2017); in addition, gibberellin (GA), temperature, vernalization and age signals can also affect plant flowering (Moon et al., 2005; Amasino, 2010; Song et al., 2013; Teotia and Tang, 2015). These signals are not independent. In *Arabidopsis*, they integrate related signals to regulate plant flower formation through integrators *FLOWERING LOCUS T (FT)*, *SUPPRESSOR OF OVEREXPRESSION OF CONSTANS 1 (SOC1)*, *LEAFY (LFY)*, etc. (Blazquez et al., 1998; Kardailsky et al., 1999; Lee et al., 2000).

In *Arabidopsis*, the GA signal mainly regulates the flower formation of plants through the interaction with the photoperiod signal and regulates the expression of *FT* under the conditions of LD and SD (Osnato et al., 2012; Song et al., 2012). In leaves, MYB-type transcription factor ASYMMETRIC LEAVES positively regulates the expression of GA biosynthesis gene *GA20ox1* (Song et al., 2012). AS1 forms a complex with CO protein and regulates FT expression by directly binding to the FT promoter (Song et al., 2012). As a central inhibitor of the GA signaling pathway, DELLA has been proven to interact with many transcription factors in leaves and stem tips and regulate their activities, thereby regulating plant flowering (Bao et al., 2020). For example, under long-day conditions, DELLA directly binds to the CCT domain of the CO protein and sequentially separates CO from the binding of the FT promoter, thereby down-regulating the expression of *FT* (Wang et al., 2016; Xu et al., 2016). In addition, DELLA can inhibit the interaction between CO and Nuclear factor Y (NF-Y) subunit B (NF-YB), and DELLA can also regulate the expression of *FT* through the interaction between PHYTOCHROME INTERACTING FACTOR 4 (PIF4) and MYC3 (de Lucas et al., 2008; Xu et al., 2016; Bao et al., 2019, 2020).

In addition, GA can directly promote flowering by up-regulating flowering integrons *LFY*, *SOC1* and *AGAMOUS-LIKE 24 (AGL24)* independently of the photoperiod pathway (Blazquez and Weigel, 2000; Moon et al., 2003; Hisamatsu and King, 2008; Liu et al., 2008). Hou et al. (2014) found that GA signal can regulate the expression of *SOC1* through epigenetic modification mediated by NF-Y complex. Under short-day conditions, the promoter activity of *LFY* gradually increased during vegetative growth, and GA enhanced the promoter activity and accelerated plant flowering (Blazquez et al., 1998). GA mainly regulates miR159 by inhibiting the expression of DELLA protein, thereby regulating the transcription of downstream *MYB33*, ultimately regulating the expression of *LFY*, and regulating the floral transformation of plants (Blazquez and Weigel, 2000; Gocal et al., 2001; Achard et al., 2009; Davis, 2009). DELLA may recruit different SPLs to target various downstream target genes, so that GA can play a role in different development environments (Bao et al., 2020). In addition, GA signal can regulate the expression of *WRKY12, WRKY13* and *WRKY75* genes through DELLA protein to regulate plant flower formation (Li et al., 2016; Zhang et al., 2018). In summary, the role of GA signal in the regulation of plant flower formation is very important and complex, but there are relatively few studies on gibberellin-mediated flower formation in woody fruit trees.

Loquat (Eriobotrya japonica Lindl.) is an evergreen fruit tree, which belongs to the Maloideae subfamily of the Rosaceae family which is mainly planted in subtropical regions. In Rosaceae, the flowering transition time and flowering time usually occur in different years (flower buds differentiate in summer and autumn, and the flower buds bloom in the spring of the second year after dormancy), including apples, pears, and strawberries (Kurokura et al., 2013). However, the flowering transformation and flowering of loquat occur in the same year. Flower bud differentiation generally occurs from July to September, with flowering occurring between October and January of the same year (Lin, 2007). The phenomenon of autumn flowering and spring harvest of loquat is very unique among woody fruit trees. In spring, the selection of fresh fruit varieties is greatly reduced. Therefore, the market demand for fresh loquat fruits in spring is relatively high and the price is relatively high. However, in cold winters (especially extreme weather events), the newly opened loquat flowers or young fruits are very susceptible to freezing damage (Peng et al., 2021), resulting in a reduction in loquat production or even no harvest.

Recent research results show that although the start time of loquat flower bud differentiation is similar to that of apples and pears, it occurs from June to July; the difference is that the development of loquat flower buds is continuous and does not undergo dormancy, and it blooms in autumn and winter (Jiang et al., 2019c). In addition, after treating loquat plants with the exogenous hormone GA_3_, the plants are in vigorous vegetative growth and cannot form flowers, and genes such as *EjSOC1s*, *EjAP1s* and *EjLFYs* are strongly inhibited (Jiang et al., 2019a,b,c). These studies show that gibberellin can regulate the flowering of loquat by regulating the expression of genes such as *EjSOC1s*, *EjAP1s* and *EjLFYs*. In Arabidopsis, *SOC1, AP1* and *LFY* genes are all downstream of the floral regulation network, and GA regulates their expression through the inhibition or activation of other transcription factors mediated by DELLAs (Bao et al., 2020). However, the mechanism of gibberellin regulating loquat flowering is not clear. Transcriptome sequencing technology is based on exogenous sequencing technology to quickly and comprehensively understand the difference level of transcripts. The application of transcriptome sequencing technology has accelerated the gene expression profile analysis and gene identification of many plant species. In this study, transcriptome analysis was performed on the materials of the GA_3_ treatment and the control group to screen the key genes related to the regulation of loquat flowering, in order to analyze the regulation mechanism of loquat flowering.

## Materials and Methods

### Plant Materials

The loquat tissue materials involved in the experiment were taken from 12-year-old “JieFangZhong” loquat plants in the loquat plant germplasm resource nursery of South China Agricultural University (Guangzhou, China, N23°09′N, 113°20′E). The experimental plants have entered the flowering and fruiting age for several years and have grown well. Loquat trees are planted in the loquat germplasm resource nursery and grow under natural conditions.

### Exogenous GA_3_ Treatment and Sample Collection

The trees were sprayed with an aqueous solution containing 0.1% (v/v) phosphoric acid and 0.025% (v/v) Triton X-100 as a surfactant and 300 mg L^–1^ GA_3_ (Dingguo Biotechnology Co., Ltd., Guangzhou, China). Spray the control plants with a solution containing only 0.1% (v/v) phosphoric acid and 0.025% (v/v) Triton X-100. The experimental treatment method was: spraying all leaves and top buds (soaked, the leaves began to drip) every 2 weeks, from May 18th to August 10th. The differentiation time of loquat flower buds is from the end of June to the beginning of July (Jiang et al., 2019c). Accordingly, the sampling time points for the GA_3_ treatment group and the control group were set as: May 25, June 29, and August 17. Mature leaves and apical buds were used in this experiment (randomly mixed with tissue samples with the same maturity in different directions and different heights of the plant, as a biological repeat). Samples of the treatment group and the control group were taken at the same time. The sample was placed in a clean centrifuge tube that has been marked, immediately frozen and stored in liquid nitrogen, and then stored in an −80°C refrigerator until use. Three independent biological replicates (the biological replicates were from separate plants) were performed for each treatment.

### RNA Extraction and Sequencing

Total RNA were extracted following the manufacturer of the RNA Prep Pure Plant Kit (TIANGEN, China). Their purity and integrity were checked and assessed using the NanoPhotometer^®^ spectrophotometer (IMPLEN, CA, United States) and RNA Nano 6000 Assay Kit of the Bioanalyzer 2100 system (Agilent Technologies, CA, United States), respectively. Subsequently, total 1 μg RNA of each sample was used as input material for the RNA sample preparations. mRNA was purified using poly-T oligo-attached magnetic beads. First-strand and second-strand cDNA was synthesized according to the manufacturer of M-MuLV Reverse Transcriptase (RNase H^–^), and DNA Polymerase I and RNase H^–^, respectively. cDNA fragments of 250∼300 bp in length were selected and purified with AMPure XP system (Beckman Coulter, Beverly, MA, United States). Besides, the library quality was assessed on the Agilent Bioanalyzer 2100 system. The clustering of the index-coded samples was performed on a cBot Cluster Generation System using TruSeq PE Cluster Kit v3-cBot-HS (Illumina) according to the manufacturer’s instructions. After cluster generation, the library preparations were sequenced on an Illumina Novaseq platform and 150 bp paired-end reads were generated.

### Genes Annotation and Differentially Expressed Genes Analysis

Raw data (raw reads) of fastq format were firstly processed through in-house perl scripts. Clean data (clean reads) were obtained by removing reads containing adapter, reads containing ploy-N and low quality reads from raw data. Q20, Q30, GC content, and sequence duplication levels in the clean data were calculated (Supplementary Table 1). All the downstream analyses were based on the clean data with high quality. Raw reads of the RNA-seq data are uploaded to Sequence Read Archive (SRA) database of NCBI with Bioproject ID number PRJNA729650.

Reference genome and gene model annotation files were downloaded from genome website (Su et al., 2021). The building of index of the reference genome, and the alignment between clean reads and reference genome all using Hisat2 (version 2.0.5). FPKM (Fragments Per Kilobase of transcript per Million fragments mapped) of each gene was calculated by featureCounts (version 1.5.0) (Florea et al., 2013).

DEGs (Differentially expressed genes) were defined by DESeq2 R package (version 1.16.1) with an adjusted *P*-value < 0.05. The resulting *P*-values were adjusted using the Benjamini and Hochberg’s approach for controlling the false discovery rate (Benjamini and Hochberg, 1995). GO (Gene Ontology) and KEGG (Kyoto Encyclopedia of Genes and Genomes) pathways enrichment analysis of DEGs was implemented by the clusterProfiler R package. Volcano plots, Venn diagrams and heatmaps were drawed by TBtools (Chen C. et al., 2020). WGCNA (weighted gene co-expression network analysis) was performed in R with the WGCNA package (Langfelder and Horvath, 2008) and visualized the networks by Cytoscape (version 3.8.2) (Shannon et al., 2003).

## Results

### Loquat Cannot Bloom After GA_3_ Treatment

**FIGURE 1:**
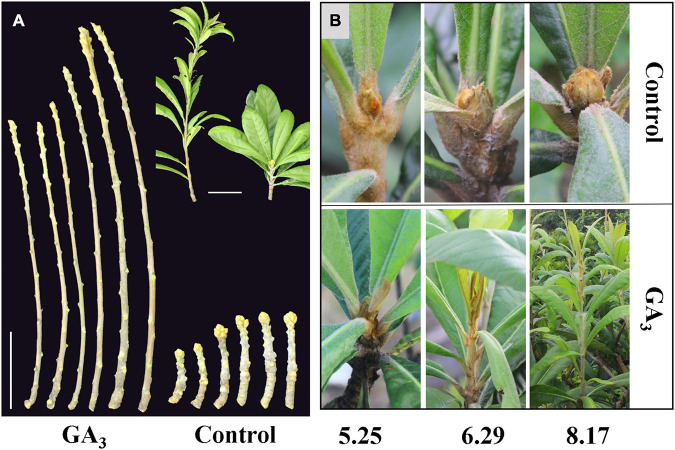
The phenotype of loquat in the GA_3_ treatment group and the control group. **(A)** GA_3_ treatment promotes the growth of stems, bars = 10 cm; **(B)** Phenotypes of samples used for transcriptome sequencing at different stages.

At 10–20 days after treating loquat plants with 300 mg L^–1^ GA_3_, vigorous vegetative growth was observed, and the stems grew rapidly. In the beginning of September when obvious inflorescence could be observed in the control group, plants in the GA_3_ treatment group were still in vigorous vegetative growth (Figures 1A,B).

### Summary Statistics of Transcriptome Sequencing

Transcriptome sequencing results showed that 41.68–56.71 Mb clean reads were obtained from the 18 samples in the control group; 41.39–61.024 Mb clean reads were obtained from the 18 samples in the GA_3_ treatment group. The GC content of the GA_3_ treatment group and the control group was similar, ranging from 45.59 to 47.08% (Supplementary Table 1). For 36 samples, 96.70% of the bases had a quality score greater than 20, and Q30 ≥ 91.39%. The sequencing data are of high quality and can meet the requirements of subsequent analysis.

### Selection of DEGs in GA_3_ Treatment Group and Control Group

**FIGURE 2:**
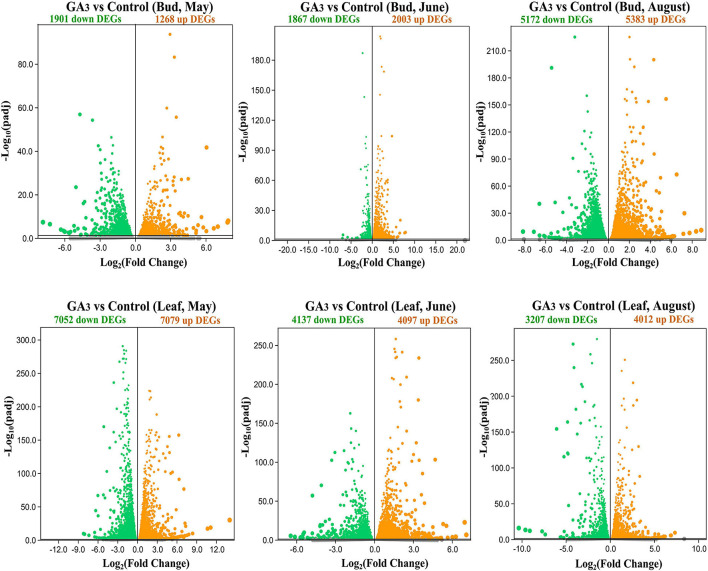
Volcano map of DEGs in the buds and leaves between the GA_3_ treatment group and the control group. Orange dots indicate up-regulated DEGs, and green dots indicate down-regulated DEGs, and gray dots indicate genes that were not differential expressed.

Compared with the control group, there were 1,901 down-regulated DEGs and 1,268 up-regulated DEGs in the buds 7 days after GA_3_ treatment on May 25th (Figure 2 and Supplementary Tables 2-1, 2); 1,867 down-regulated DEGs and 2,003 up-regulated DEGs in the buds on June 29th (Figure 2 and Supplementary Tables 2-3, 4); 5,172 down-regulated DEGs and 5,383 up-regulated DEGs in the buds on August 17th (Figure 2 and Supplementary Tables 2-5, 6). In leaves, compared with the control group, there were 7,052 down-regulated DEGs and 7,079 up-regulated DEGs in the leaves 7 days after GA_3_ treatment on May 25th (Figure 2 and Supplementary Tables 3-1, 2); 4,137 down-regulated DEGs and 4,097 up-regulated DEGs in the leaves on June 29th (Figure 2 and Supplementary Tables 3-3, 4); 3,207 down-regulated DEGs and 4,012 up-regulated DEGs in the leaves on August 17th (Figure 2 and Supplementary Tables 3-5, 6).

The results showed that compared with the control group, the number of DEGs in the apical buds of the GA_3_ treatment group increased rapidly from June 29th to August 17th (Figure 2). It implies that during this period, GA_3_ regulates the flower bud differentiation of loquat by up-regulating or down-regulating the expressions of a large number of flowering-related genes. In comparison, highly number of DEGs were expressed in May in leaves before the bud differentiation of loquat (Figure 2).

### Functional Annotation and Expression Patterns of DEGs

**FIGURE 3:**
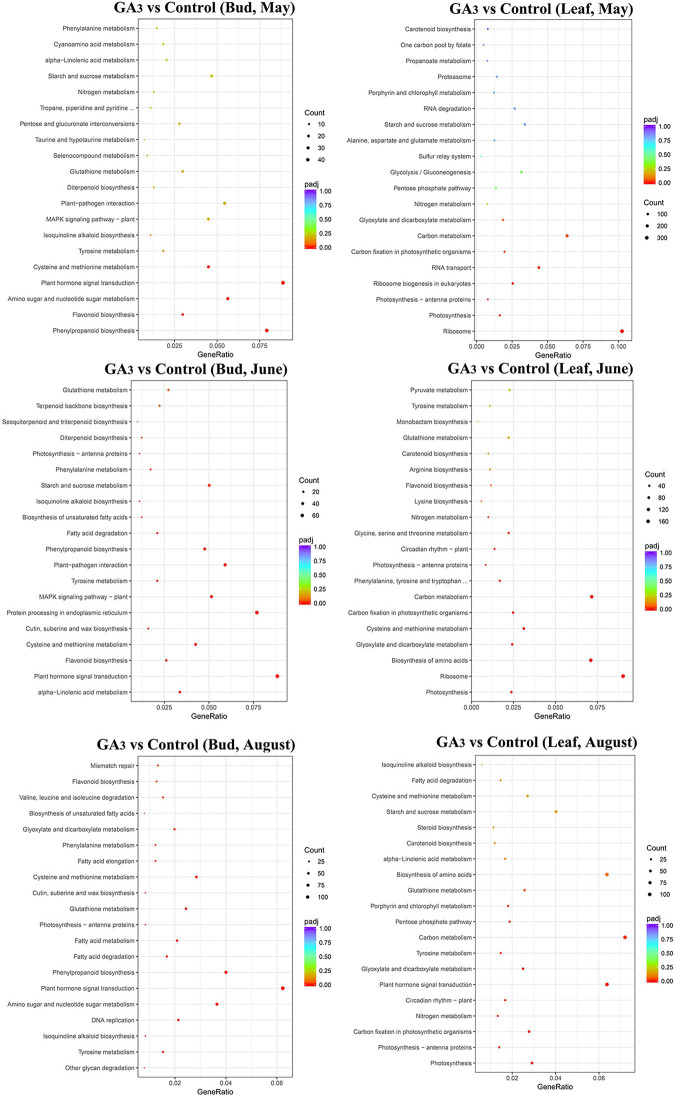
KEGG analysis of DEGs in different tissues of GA_3_ treatment group and control group at different periods.

In order to explore how loquat responds to GA_3_ signals, GO and KEGG enrichment analysis were performed on DEGs the compare between GA_3_ treatment and control of buds and leaves. GO enrichment showed that DEGs of buds mainly involved in biological signal binding and catalytic activity, such as heme binding, tetrapyrrole binding, hydrolase activity, acting on glycosyl bonds, etc. (Figure 3 and Supplementary Tables 4-1, 2, 3). In leaves, DEGs mainly involved in metabolism, transcriptional activity, and biosynthesis, such as peptide metabolic process, amide biosynthetic process, peptide biosynthetic process, nucleic acid binding transcription factor activity, transcription factor activity (sequence-specific DNA binding), etc. (Supplementary Figure 1 and Supplementary Tables 4-4, 5, 6).

The KEGG annotation shows that the 841 DEGs in the buds 7 days after GA_3_ treatment were enriched in 110 KEGG pathways, among which plant hormone signal transduction (47 genes, 5.59%), phenylpropanoid biosynthesis (42 genes, 4.99%), and amino acids biosynthesis (35 genes, 4.16%) were significantly enriched pathways (Figure 3 and Supplementary Table 5-1). Total of 1,278 DEGs in the buds on June 29th were enriched in 115 KEGG pathways, among which plant hormone signal transduction (70 genes, 5.48%), protein processing in endoplasmic reticulum (61 genes, 4.77%), and plant-pathogen interaction (47 genes, 3.68%) were significantly enriched pathways (Figure 3 and Supplementary Table 5-2). Total of 3,327 DEGs in the buds on August 17th were enriched in 120 KEGG pathways, among which carbon metabolism (125 genes, 3.76%), ribosome (125 genes, 3.76%), and plant hormone signal transduction (124 genes, 3.73%) were significantly enriched pathways (Figure 3 and Supplementary Table 5-3).

In leaves, KEGG annotation shows that the 4,627 DEGs in the leaves on May 25th were enriched in 120 KEGG pathways, among which ribosome (302 genes, 6.53%), carbon metabolism (189 genes, 4.08%), biosynthesis of amino acids (153, 3.31%), and plant hormone signal transduction (137 genes, 2.96%) were significantly enriched pathways (Figure 3 and Supplementary Table 5-4). A total of 3,059 DEGs in the leaves on June 29th were enriched in 120 KEGG pathways, among which ribosome (171 genes, 5.59%), carbon metabolism (136 genes, 4.45%), biosynthesis of amino acids (135 genes, 4.41%), and plant hormone signal transduction (89 genes, 2.91%) were significantly enriched pathways (Figure 3 and Supplementary Table 5-5). 2,395 DEGs in the leaves on August 17th were enriched in 119 KEGG pathways, among which carbon metabolism (105 genes, 4.38%), plant hormone signal transduction (93 genes, 3.88%), biosynthesis of amino acids (93 genes, 3.88%), and ribosome (84 genes, 3.51%) were significantly enriched pathways (Figure 3 and Supplementary Table 5-6).

The results showed that DEGs were mainly enriched in the plant hormone signal transduction pathway after GA_3_ treatment, which also indicated that after GA_3_ treatment, loquats mainly responded to GA_3_ signals through these DEGs, and ultimately participated in the regulation of loquat flower bud differentiation.

**FIGURE 4:**
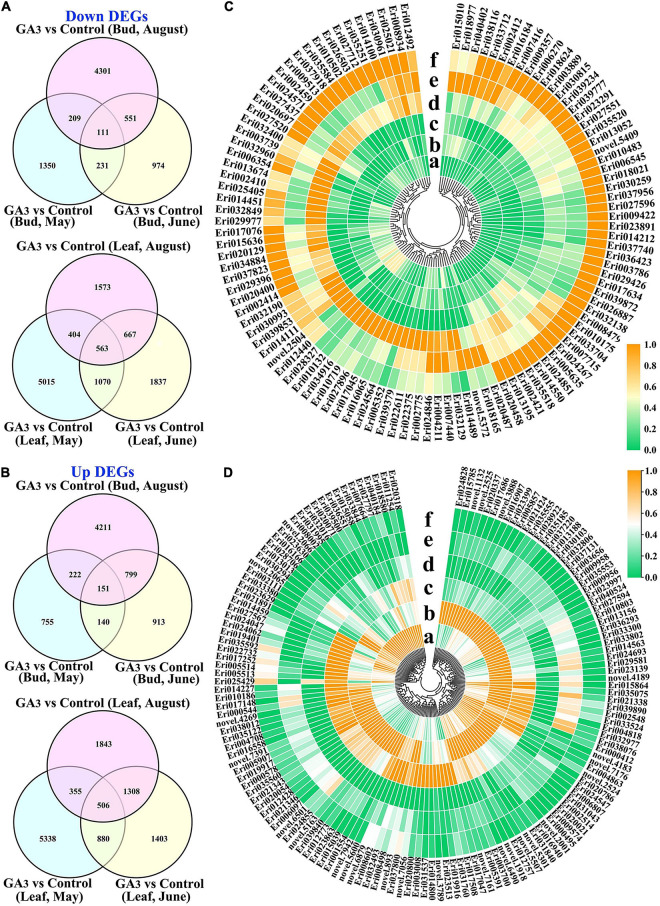
DEGs analysis of loquat leaves and buds transcriptome datasets. **(A)** Venn diagrams of DEGs that are down-regulated in leaves and buds at different stages; **(B)** Venn diagrams of DEGs that are up-regulated in leaves and buds at different stages; **(C)** The expression patterns of 111 down-regulated DEGs in buds; **(D)** The expression patterns of 151 up-regulated DEGs in buds. a, b, and c respectively represent the material on May 25th, June 29th, and August 17th after GA3 treatment; d, e, and f represent the control group materials on May 25th, June 29th, and August 17th, respectively.

In order to further understand the expression patterns of genes related to flower bud differentiation, we performed a cluster analysis on the selected DEGs. It was revealed that after GA_3_ treatment, 111 DEGs were down-regulated in the three stages of buds (Figures 4A,C and Supplementary Table 6-1) and 151 DEGs were up-regulated (Figures 4B,D and Supplementary Table 6-2). In addition, we found in the leaves that 563 DEGs were down-regulated in the three stages after GA_3_ treatment (Figure 4A and Supplementary Table 6-3), and 506 DEGs were up-regulated (Figure 4B and Supplementary Table 6-4).

Through the analysis of all down-regulated and up-regulated DEGs in buds with annotation results, it was found that 44 down-regulated DEGs belonged to 27 gene families (Supplementary Figure 2A); while the 58 up-regulated DEGs belonged to 28 gene families (Supplementary Figure 2B) including AP2, bZIP, F-box, MYB, WRKY and other gene family genes. The flower buds of loquat cannot differentiate after GA_3_ treatment, which also implies that the down-regulated DEGs after treatment are possibly positive-regulatory genes involved in loquat flowering, and these up-regulated DEGs may be negative-regulatory genes for loquat flowering.

### Expression Analysis of DELLA-Mediated GA Signal Regulatory Network in Loquat Flowering

**FIGURE 5:**
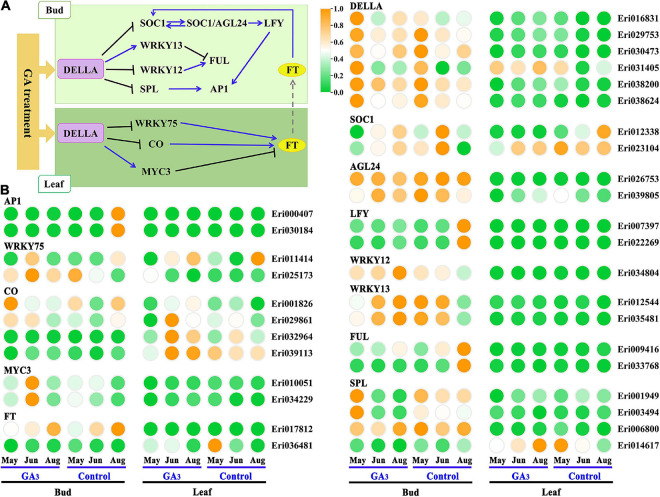
Expression analysis of homologous genes from DELLA-mediated GA signal regulatory pathway in loquat. **(A)** DELLA-mediated GA signal regulation pathway in *Arabidopsis*. **(B)** The expression pattern of homologous genes from the GA pathway in loquat leaves and buds.

In *Arabidopsis thaliana*, the GA pathway genes involved in the regulation of flower formation mainly include *DELLA, CO, MYC3, WRKY75, WRKY12, WRKY13, SOCl, SPL, FT, AGL24, LFY, FUL, AP1*, etc. (Bao et al., 2020). Thirty-three homologous genes in loquat were obtained through sequence alignment by blast the transcripts of loquat and coding sequence of *Arabidopsis thaliana* genome (Figure 5 and Supplementary Table 7). Based on the expression patterns of these homologous genes in the GA_3_ treatment group and the control group, thirteen candidate genes, including *EjWRKY75* (Eri011414), EjFT (Eri036481), EjSOC1 (Eri012338, Eri023104), EjAGL24 (Eri026753), EjSPL (Eri001949, Eri003494), EjLFY (Eri007397, Eri022269), EjFUL (Eri009416, Eri033768), EjAP1 (Eri000407, Eri030184), were highly expressed in control group than GA3 treatment group in buds, and may be involved in the positive regulation of flower development. Besides, eleven candidate genes, including EjDELLA (Eri016831, Eri029753, Eri030473, Eri031405, Eri038200, Eri038624), EjMYC3 (Eri010051, Eri034229), EjWRKY12 (Eri034804), EjWRKY13 (Eri012544, Eri035481), were highly expressed in GA_3_ treatment group than control group in buds, and may be involved in the negative regulation of flower development. However, we found that the expressions of *EjCOs* in leaves did not decrease but increased after GA_3_ treatment. It suggests that the *EjCOs* in loquat may be mainly regulated by photoperiod, rather than regulating loquat flower development by responding to GA_3_ signals.

### Co-expression Network Analysis of Genes Involved in the Regulation of Flower Formation

*EjSOC1s* play an active role in the flowering process of loquat, *EjAP1s* and *EjLFYs* can be used as marker genes for loquat flowering regulation (Jiang et al., 2019c). Our experimental results also further confirmed this conclusion. In Arabidopsis, SOC1 or LFY activates the expressions of floral meristem identity genes *LFY, AP1*, and *FUL* to initiate flower bud differentiation (Blazquez et al., 1998; Blazquez and Weigel, 2000).

**FIGURE 6:**
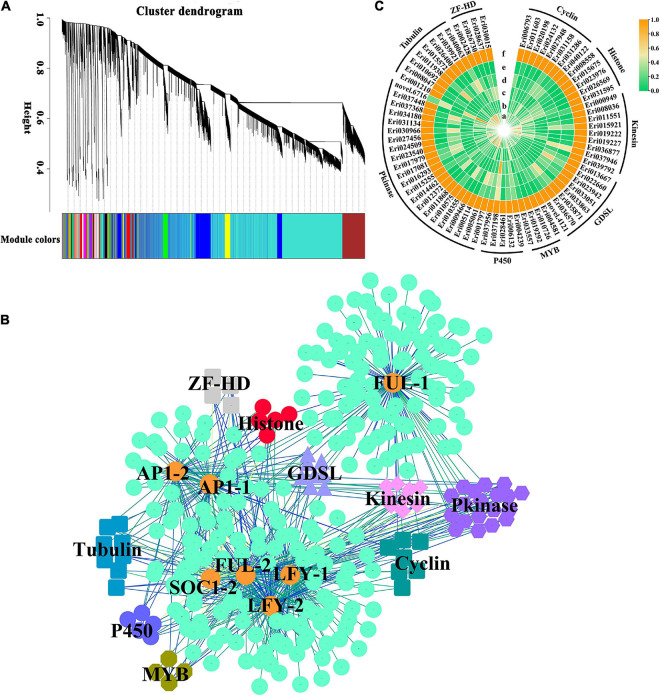
WGCNA co-expression analysis of homolog genes from DELLA-mediated GA signal regulatory pathway in loquat. **(A)** Module trait relationships in the GA_3_ treatment group and the control group. **(B)** The co-expression network connecting the key genes in DELLA-mediated GA signal regulatory pathway with candidate genes in brown module. Expression associations between homolog genes (orange circle) from GA pathway and candidate genes (other shapes except orange circle) are indicated with colored lines according to weight. **(C)** The expression pattern of candidate genes in loquat buds.

In order to further understand the regulatory network of loquat flowering genes and the regulatory relationship of flowering-related genes, WGCNA analysis was carried out to investigate the co-expression networks of DEGs in the transcriptome data, in which all the co-expressed genes were connected to each other with varying association strengths. DEGs are clustered into 27 modules according to the expression patterns (Figure 6A), interestingly, *EjSOC1-2* (Eri023104), EjLFY-1 (Eri007397), EjLFY-2 (Eri022269), EjFUL-1 (Eri009416), EjFUL-2 (Eri033768), EjAP1-1 (Eri000407) and EjAP1-2 (Eri030184) were assigned to the brown module, except for EjSOC1-1 (Eri012338) in the yellow module (weight < 0.1). Further analysis of the seven genes in the brown module revealed that 330 genes were co-expressed with them (Figure 6B and Supplementary Table 8), belonging to 74 gene families, including Cyclin_C, Histone, Kinesin, Lipase_GDSL, MYB, P450, Pkinase, Tubulin, ZF-HD_dimer gene family (Figure 6C). It showed that their expression patterns are similar to that of *EjSOC1-2, EjLFYs, EjFULs, EjAP1s*, and they are all inhibited by GA_3_ treatment. It implied that these genes may play a positive regulatory role in the flowering of loquat. Therefore, these data provide new directions and useful candidate genes for research on loquat flowering regulation.

## Discussion

The Rosaceae family contains many fruit crop species, such as apple, pear, peach, strawberry and loquat. However, the unique flowering time of autumn flowering and spring fruit ripening of cultivated loquat suggests that it may have a flowering regulation system different from other woody plants in Rosaceae family during the evolutionary process. Although the flowering habit of loquat is very special, there are few reports about it. So far, several flowering-related genes have been cloned from cultivated loquat, including *EjAP1, EjAP3, EjFT, EjLFY, EjSOC1, EjSVP, EjSPL, EjFRI* and *EjTFL1* (Esumi et al., 2005; Liu et al., 2013, 2017; Reig et al., 2017; Jiang et al., 2019a,b,c; Chen W. et al., 2020). *EdCO, EdGI, EdFT* and *EdFD* have been cloned from wild loquat *Eriobotrya deflexa* Nakai forma *koshunensis* (Zhang L. et al., 2016; Zhang et al., 2019). However, the loquat floral regulation network is still unclear. This study enriched the loquat floral regulation network resources, which can provide an important reference for further analysis of the molecular mechanism of loquat flowering, and also provide a theoretical basis for the later research on the floral regulation of Rosaceae.

The two hormone systems “gibberellin” and “florigen” (FT) play a key role in crop yield and quality (Eshed and Lippman, 2019). Previous studies have shown that GA_3_ treatment can inhibit the flower bud induction of nectarine, sweet cherry, mango, citrus and apple (García-Pallas et al., 2001; Lenahan et al., 2006; Nakagawa et al., 2012; Goldberg-Moeller et al., 2013; Zhang S. et al., 2016). The *miFT* in mangoes can regulate the flowering by responding to GA signals (Nakagawa et al., 2012); in citrus (*Citrus reticulata* Blanco × *Citrus temple* Hort. ex Y. Tanaka), *FT, AP1* and a few flower-organ-identity genes are inhibited by GA, but GA promotes the expression of *LFY* (Goldberg-Moeller et al., 2013); The expression levels of *MdSPLs, MdFT, MdSOC1* and *MdAP1* genes in apples are all inhibited by the GA_3_ treatment (Zhang S. et al., 2016). In this study, we also found similar conclusions to that from these woody fruit trees. For example, GA_3_ treatment inhibited the flowering of loquat, and the expressions of genes such as *EjFT, EjSOC1, EjSPL*, and *EjAP1* were inhibited. But different from the expression in citrus, the expression of *EjLFY* in loquat was inhibited by GA_3_. Our results also showed that some genes are different from the expression patterns in model plants, such as *EjMYC3*, which mainly plays a regulatory role in Arabidopsis leaves, while the difference in expression of *EjMYC3* in loquat after GA_3_ treatment mainly occurs in buds. There is little difference in expression in leaves, suggesting that it mainly plays a role in regulating flowering in buds. In addition, we found that the expressions of several *EjCO* genes did not decrease but increased after GA_3_ treatment. We speculated that it may be mainly through response to photoperiod signals to regulate flower formation, rather than GA signals.

In this study, by analyzing all the down-regulated and up-regulated DEGs in the apical buds of different periods, some candidate genes that may participate in loquat flower bud differentiation were screened, including AP2, bZIP, F-box, MYB, WRKY and other gene families (Supplementary Figure 2). In addition, through WGCNA analysis, candidate genes co-expressed with *EjSOC1-2, EjLFYs, EjFULs, EjAP1s*, and possibly involved in loquat flower bud differentiation, including Cyclin_C, Histone, Kinesin, Lipase_GDSL, MYB, P450, Pkinase, Tubulin, ZF- HD_dimer gene family. The discovery of these candidate genes has brought great convenience to the subsequent study of loquat flower formation. Future work will focus on verifying and analyzing the functions and mechanisms of these candidate genes in the formation of loquat flowers.

**FIGURE 7:**
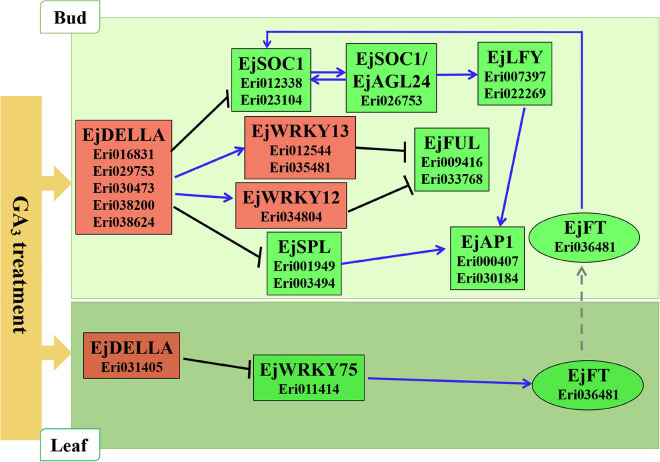
Hypothetical model of loquat flowering regulation.

Referring to the DELLA-mediated GA signal regulation network diagram in Arabidopsis (Figure 6A), we constructed a hypothetical model for loquat flowering regulation network based on the expression patterns of related homologous genes in loquat (Figure 7). As follows: In leaves, GA_3_ promotes the expression of DELLA family genes (Eri031405) to inhibit the expression of EjWRKY75 (Eri011414), and then EjWRKY75 down-regulates the expression of EjFT (Eri036481), and finally the EjFT protein is transported to SAMs to take effect. In SAMs, GA3 promotes the expression of DELLA family genes (Eri016831, Eri029753, Eri030473, Eri038200, and Eri038624). On the one hand, DELLA family genes inhibit the expression of EjFUL (Eri009416, Eri033768) by promoting the expression of EjWRKY12 (Eri034804) and EjWRKY13 (Eri012544, Eri035481). On the other hand, DELLA family genes inhibited the expression of flower-specific genes EjLFY (Eri007397, Eri022269) and EjAP1 (Eri000407, Eri030184) by inhibiting the expression of EjSOC1 (Eri012338, Eri023104), EjAGL24 (Eri026753) and EjSPL (Eri001949, Eri003494). Finally, the flower development of loquat was inhibited.

## Data Availability Statement

The original contributions presented in the study are publicly available in NCBI using accession number PRJNA729650.

## Author Contributions

YJ, YL, and FX designed the research. YJ and FX mainly performed the research. YG, JP, WS, YY, XY, CZ, MW, and SL contributed reagents, materials, and analysis tools. YJ, FX, and ZP wrote the manuscript. ZP and FX revised and approved the manuscript. All authors contributed to the article and approved the submitted version.

## Conflict of Interest

The authors declare that the research was conducted in the absence of any commercial or financial relationships that could be construed as a potential conflict of interest. The handling editor declared a past collaboration with one of the author YJ.

## Publisher’s Note

All claims expressed in this article are solely those of the authors and do not necessarily represent those of their affiliated organizations, or those of the publisher, the editors and the reviewers. Any product that may be evaluated in this article, or claim that may be made by its manufacturer, is not guaranteed or endorsed by the publisher.
