# Supplementary material for: Gibberellin Induced Transcriptome Profiles Reveal Gene Regulation of Loquat Flowering
Source: Front Genet. 2021 Sep 10;12:703688. doi: 10.3389/fgene.2021.703688 (PMC8460860; doi:10.3389/fgene.2021.703688)
Supplement: Supplementary file 9 [file Data_Sheet_1.docx]

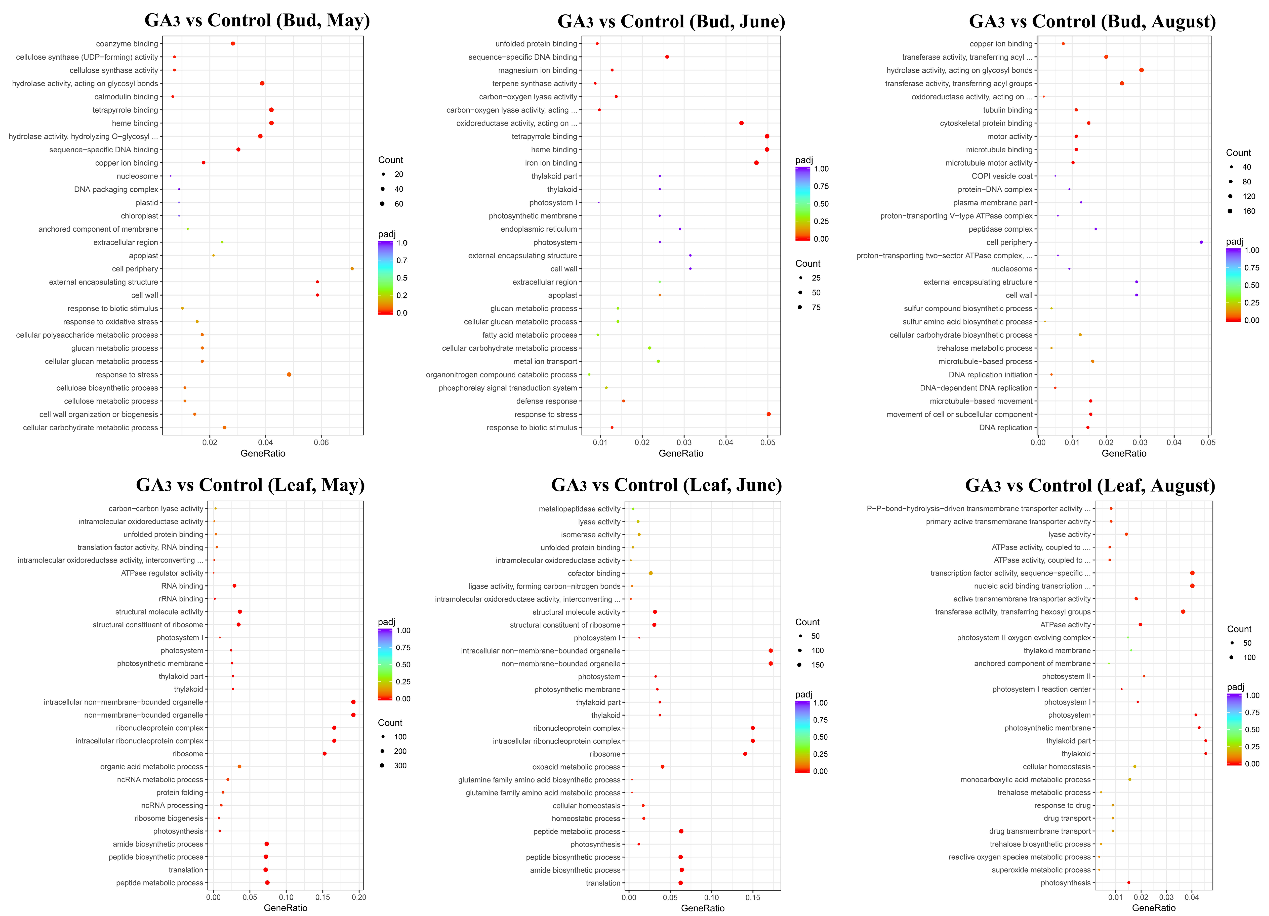


Figure S1. GO analysis of DEGs in different tissues of GA_3_ treatment group and control group at different periods


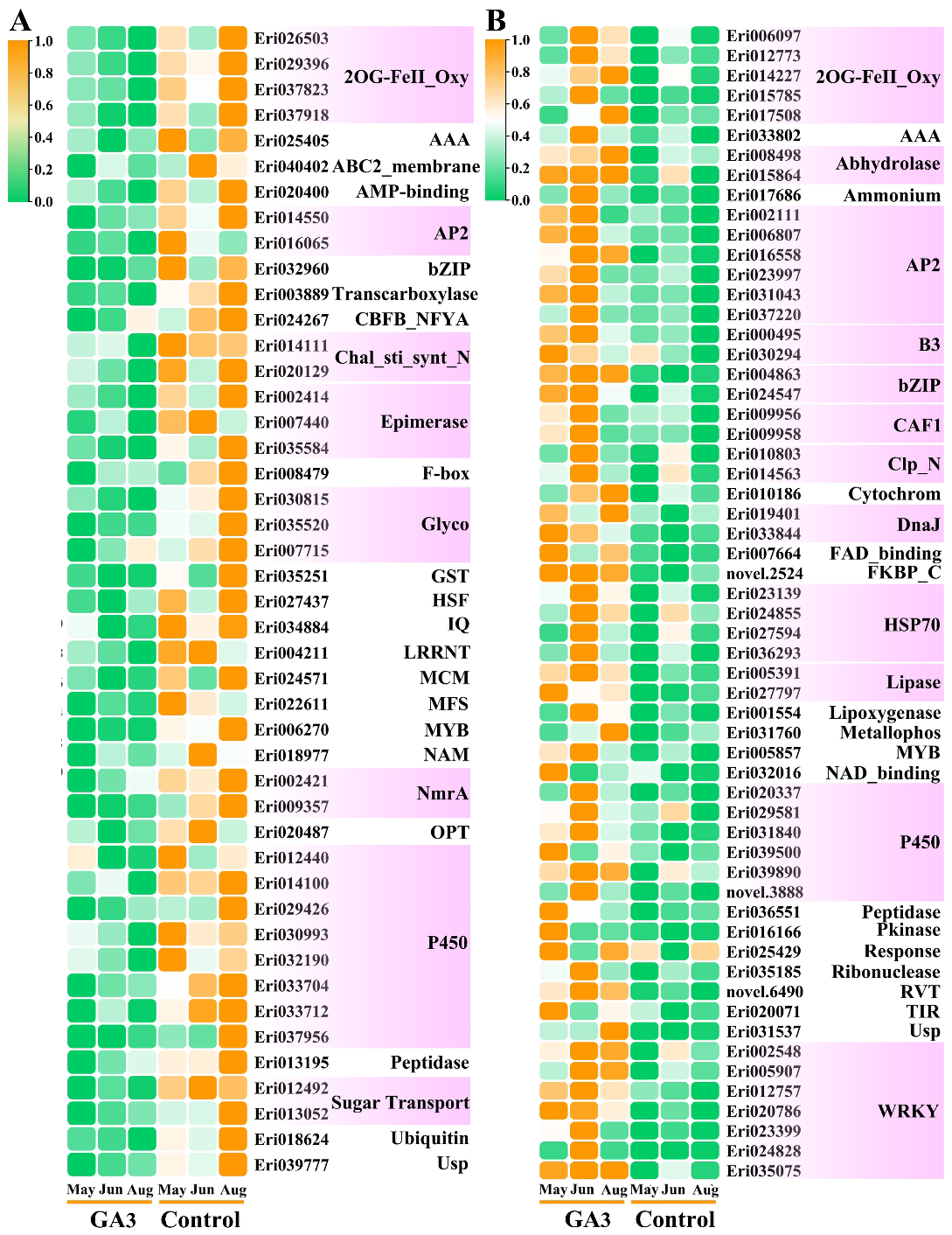


Figure S2. DEGs analysis of loquat buds transcriptome datasets. (A) The annotation of 111 down-regulated DEGs (Figure 4C) in buds; (B) The annotation of 151 up-regulated DEGs (Figure 4D) in buds.
